# Genomic Analyses of Human Sapoviruses Detected over a 40-Year Period Reveal Disparate Patterns of Evolution among Genotypes and Genome Regions

**DOI:** 10.3390/v12050516

**Published:** 2020-05-07

**Authors:** Kentaro Tohma, Michael Kulka, Suzie Coughlan, Kim Y. Green, Gabriel I. Parra

**Affiliations:** 1 Division of Viral Products, CBER, FDA, Silver Spring, MD 20993, USA; 2 Division of Molecular Biology, CFSAN, FDA, Laurel, MD 20993, USA; Michael.Kulka@fda.hhs.gov; 3 National Virus Reference Laboratory, UC-Dublin, Dublin 4, Ireland; suzie.coughlan@ucd.ie; 4 Laboratory of Infectious Diseases, NIH, Bethesda, MD 20993, USA; kim.green@nih.gov

**Keywords:** sapovirus, calicivirus, genetic diversity, evolution, recombination, norovirus

## Abstract

Human sapovirus is a causative agent of acute gastroenteritis in all age groups. The use of full-length viral genomes has proven beneficial to investigate evolutionary dynamics and transmission chains. In this study, we developed a full-length genome sequencing platform for human sapovirus and sequenced the oldest available strains (collected in the 1970s) to analyse diversification of sapoviruses. Sequence analyses from five major genotypes (GI.1, GI.2, GII.1, GII.3, and GIV.1) showed limited intra-genotypic diversification for over 20–40 years. The accumulation of amino acid mutations in VP1 was detected for GI.2 and GIV.1 viruses, while having a similar rate of nucleotide evolution to the other genotypes. Differences in the phylogenetic clustering were detected between RdRp and VP1 sequences of our archival strains as well as other reported putative recombinants. However, the lack of the parental strains and differences in diversification among genomic regions suggest that discrepancies in the phylogenetic clustering of sapoviruses could be explained, not only by recombination, but also by disparate nucleotide substitution patterns between RdRp and VP1 sequences. Together, this study shows that, contrary to noroviruses, sapoviruses present limited diversification by means of intra-genotype variation and recombination.

## 1. Introduction

Human sapovirus is a causative agent of acute gastroenteritis in people from all age-groups. A member of the family *Caliciviridae*, sapoviruses are non-enveloped and their viral capsid has icosahedral symmetry. Their genomes are single-stranded, positive-sense, 3’-polyadenylated RNA molecules of approximately 7.4 kb in length. The genome is organized into two open reading frames (ORFs), which are flanked by a short 5’-end and a long 3’-end non-coding regions. An ORF3 has been predicted in some sapovirus strains, but its function is still unknown [1]. The ORF1 encodes the nonstructural proteins, including the RNA dependent RNA polymerase (RdRp), and the major viral capsid protein (VP1) [1,2]. Cryo-electron microscopy and homology modeling revealed that VP1 is structurally divided into a shell (S) and protruding (P) domains; the latter further divided into P1 and P2 subdomains [3]. Based on the genetic differences of the VP1-encoding sequences, human sapoviruses are phylogenetically clustered into four genogroups (GI, GII, GIV and GV), with each genogroup further clustered into multiple genotypes [1,4]. Discrepancies in the phylogenetic clustering can occur when using different genomic regions, particularly those encoding the RdRp and VP1, and this has led to the identification of intra- and inter-genogroup recombinant strains [1,5,6,7,8,9,10,11,12,13]. Indeed, recombination has been shown to be an important mechanism of diversification for norovirus and other caliciviruses [1,14,15]. Some of the recombination events reported for sapoviruses did not clearly describe both parental strains [5,6,7,10,13], thus the “recombination signal” might be confounded by the possibility of different evolutionary diversification patterns among sapovirus genomic regions [16].

Human sapovirus symptoms can include diarrhea, vomiting, nausea, stomach cramps, and myalgia. Sapovirus-related symptoms are usually mild, and can be resolved within a few days; however, severe disease has been reported in vulnerable individuals and can result in hospitalization [11,16]. Sapovirus transmission occurs by person-to-person contact and/or by consumption of contaminated water, soil, or food [1,17]. Similar to norovirus, gastroenteritis outbreaks associated to sapoviruses occur in semi-enclosed settings, such as childcare facilities, schools, nursing homes, and catering receptions [18,19].

Genomic analyses for evolutionary studies [20], epidemiological investigations [21], and transmission tracking of viral infections [22] have proven beneficial to control and prevent infections and mitigate the burden on patient care [23,24]. The use of whole, versus short, genome sequences can enhance those studies, particularly in viruses that can rapidly acquire mutations [25,26]. The application of metagenomic analyses using whole genome sequencing is increasing in use in clinical settings, because of the power and potential for obtaining longer nucleotide sequence reads of a pathogen’s genome [27]. However, in most cases, the performance of these metagenomic analyses are greatly affected by the concentration of the viral contaminant and the complexity of the food and/or water matrix [28]. Different alternatives have been implemented in recent years, such as the enrichment of viral particles and random or targeted amplification of viral genomes, to improve the performance of the metagenomic approaches for viral diagnostics and outbreak investigations [29,30,31,32]. The aim of this study was to develop a simplified full-length viral genome sequencing platform for analyses of human sapovirus genomes. This new platform was used to sequence archival sapovirus-positive samples dating from the 1970s, and together with those sequences available in public databases were used to characterize the evolutionary dynamics of human sapoviruses. A better understanding of the evolutionary process that drives viral diversification could provide insights into the development of therapeutic and control strategies against these viruses.

## 2. Materials and Methods

### 2.1. Fecal Samples Positive for Human Sapovirus

Fifteen human fecal samples positive for human sapovirus were tested for full-length genome PCR amplification, sequencing, and evolutionary analysis. The samples were collected as part of a study conducted by the World Health Organization in different countries during 1976–1979 [33]. The retrospective research use of the samples in this study was approved by the NIAID Institutional Review Board. Samples were de-identified, and a waiver of informed consent was granted for this use. Four additional human fecal samples obtained from the National Virus reference Laboratory-UC Dublin confirmed positive for human sapovirus, and identified as GV.1 (GenBank accession MK291480), were tested for full-length genome PCR amplification.

### 2.2. Full-Length Viral Genome PCR and Sequencing

Sapovirus RNA genome was extracted from 10% stool suspensions using the MagMax Viral RNA Isolation Kit (Thermo Fisher Scientific, MA, USA). Viral RNA genome was retrotranscribed to complementary DNA (cDNA) using the Maxima Minus First Strand cDNA Synthesis Kit (Thermo Fisher Scientific). The cDNA reaction was performed immediately after viral RNA extraction to reduce damage of the long viral RNA molecules by a freeze and thaw cycle. The annealing reaction (65 °C, 5 min then hold at 5 °C) contained 5 µL of extracted sample RNA, 2 µL 100 µM of the primer TX30SXN [34], 1 µL 10 mM dNTPs and volume adjusted to 14 µL final with DNase/RNase free (DNF) water. Following the annealing step, a single 6 µL mixture containing 4 µL of 5× RT first-strand synthesis buffer, 0.1 µL Max H minus Enzyme Mix and 1.9 µL DNF water was added to each annealing reaction while on ice. The RT reaction was completed by incubation at 50 °C for 1 h, 85 °C for 5 min, then held at 4 °C and/or stored at −80 °C until use in the full-length viral genome PCR reaction.

The full-length viral genome amplification was performed using a similar protocol, as described for noroviruses [35]. Briefly, the SequalPrep Long PCR Kit (Thermo Fisher Scientific) was used with 5 ul of viral cDNA and 2 µL of each (10 µM) primer pSapo27 (5’ GTGATTGGTTAGTATGGCTTCCAAGCC 3’) and TX30SXN [34]. The complete PCR reaction was run in 1% agarose gel, and the resulting full-length viral genome amplicons (~7.4 kbp) were excised and purified using the Qiagen Gel Extraction Kit (Qiagen, CA, USA). The recovered amplicons were quantified using the Qubit dsDNA HS Assay Kit (Thermo Fisher Scientific) and subjected to next-generation sequencing (NGS). The library for NGS was prepared using the Nextera DNA Flex Library Prep Kit (Illumina, CA, USA), and the paired-end 2 × 150 bp sequence reads were obtained using the MiSeq system (Illumina). Reads were quality-filtered (base quality score ≥ 20) and mapped against reference genomes to reconstruct their consensus sequence using HIVE platform [36], as described previously [37]. Newly obtained nearly full-length sapovirus sequences were deposited in GenBank (accession numbers MN794205–MN794218).

### 2.3. Sequence Analyses

To investigate the phylogenetic relationship and evolutionary dynamics of human sapoviruses, sequences from archival samples were analyzed with publicly available sequences. A dataset of nearly full-length genome sequences (>7000 nucleotide, *n* = 139), the VP1-encoding nucleotide sequences (>1600 nucleotide, *n* = 233), and the RdRp-encoding sequences (>650 nucleotide, *n* = 166) from human sapovirus genotypes were obtained from GenBank on January 31, 2019 (Table S1). Sequences from each genogroup/genotype were separately aligned using MUSCLE [38]. The phylogenetic relationship of the archival strains and reference strains from GenBank was analyzed using the RdRp- and the VP1-encoding nucleotide sequences using maximum-likelihood method implemented in MEGA7 [39]. The best substitution model of each dataset was selected, as per the Bayesian information criterion. The reconstructed phylogenetic trees were visualized using FigTree v1.4.3. The clock-like signal of the RdRp- and VP1-encoding nucleotide sequences were analyzed using inferred maximum-likelihood trees using TempEst v1.5 [40]. The root-to-tip divergence plot was constructed at the genotype level and the best-fitting root option was used to minimize the sum of the squared residuals. The evolutionary rate of the RdRp- and VP1-encoding nucleotide sequences was estimated using the Bayesian Markov chain Monte Carlo (MCMC) framework, as implemented in BEAST v1.8.4 [41]. All the analyzed genotypes showed strong clock-likeness and thus, we applied the strict clock model. The SRD06 model was used for estimating the substitution process of the sequences [42]. The population size was assumed to be constant throughout their evolutionary history. The MCMC runs were performed until the convergence of all the parameters was confirmed by visual inspection and effective sample size (>200 in all parameters), using Tracer v1.6 (http://tree.bio.ed.ac.uk/software/tracer/). The first 10% of the logs from the MCMC runs were removed as a burn-in, before summarizing the posterior values. The posterior values of the substitution rate were summarized using GraphPad Prism v7. To investigate the pattern of accumulation of amino acid substitutions in the VP1, the pairwise amino acid differences and the timespan of detection for each paired-strain were calculated using a Python script described elsewhere [35] and visualized using GraphPad Prism v7. Shannon entropy values were calculated using the Shannon Entropy-One tool as implemented in Los Alamos National Laboratory (www.hiv.lanl.gov). Entropy values for each amino acid position on the ORF1 and 2 were plotted and subjected to one-way ANOVA with Tukey’s post-hoc multiple comparison test using GraphPad Prism v7. Entropy values for each nucleotide position at the RdRp-VP1 boundaries were calculated for human sapovirus and human norovirus for comparison. For human noroviruses, 1669 (nearly) full-length genome sequences from human norovirus GI and GII strains were obtained from GenBank on August 23, 2019. Sequences were multiple-aligned using MAFFT [43], and trimmed into the VP1-encoding region or approximately 350 nucleotides of the RdRp-VP1 junction region for the entropy analyses. Finally, the recombination signal of the archival strains (GI.1/HK37/1977 and GII.4/T003/1976) was assessed using its nearly full-length genome sequences and SimPlot v3.5.1 [44]. The nucleotide similarity (window-size 200 nucleotide and step-size 20 nucleotide) was calculated using Kimura 2-parameter model and was plotted using GraphPad Prism v7. 

## 3. Results and Discussion

**Figure 1:**
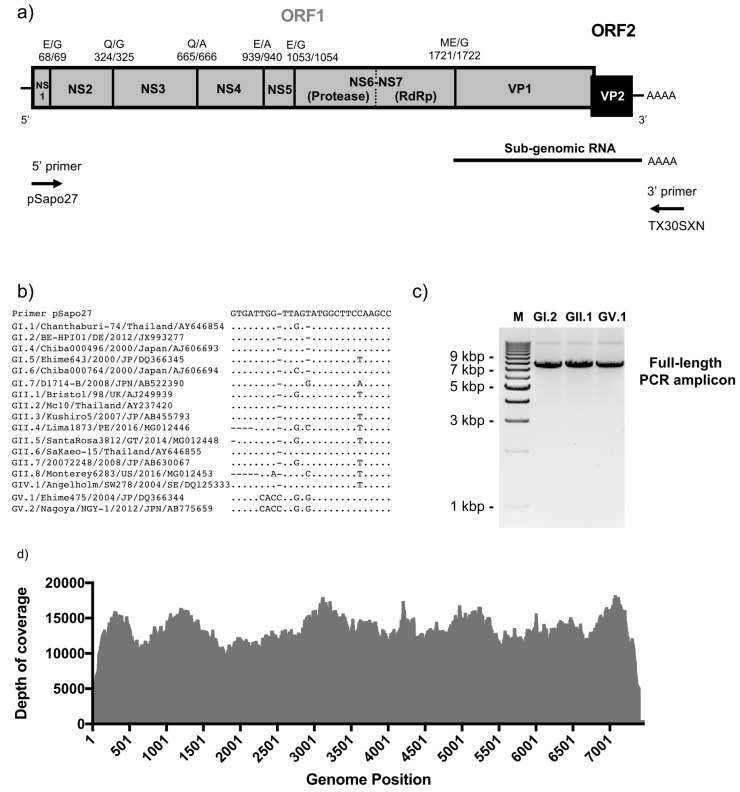
Full-length sapovirus genome PCR amplification and sequencing. (**a**) Sapovirus genome structure and primers pSapo27 and TX30SXN designed for one-step RT-PCR. Open reading frames (ORFs) and predicted cleavage sites are indicated on top. Amino acid positions are based on GI.1/Manchester/93 (X86560) strain (**b**) Alignment of 5’-primer (pSapo27) with human sapovirus genomes available in public repositories. (**c**) Agarose gel electrophoresis of full-length genome PCR amplicons of sapoviruses representing each of the genogroups GI, GII and GV. (**d**) Graph showing the depth of reads coverage in nearly-full length sapovirus genome sequences obtained from one of the archival samples (C102) in our study.

**Figure 2:**
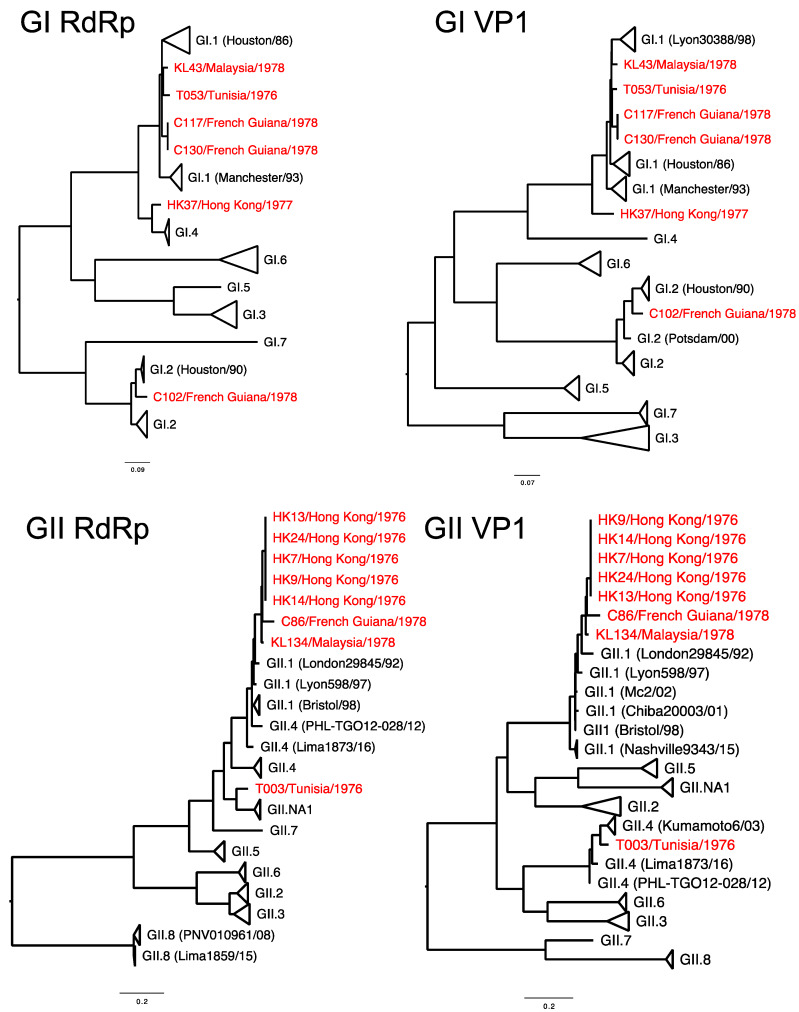
Phylogenetic tree of sapovirus GI and GII strains, circulating since 1976. The maximum likelihood phylogenetic trees were constructed using Kimura-2 parameter model with bootstrap values of ≥70. Samples analyzed in this study are highlighted in red. Sequences for partial RdRp (viral polymerase, >650 nucleotide length) and nearly full-length VP1 (≥1575 nucleotide length) were separately analyzed for genotyping and detection of possible recombinants.

The aim of this work was to develop a simplified full-length viral genome sequencing platform for analyses of human sapovirus genomes and evolution. To minimize the cost of sequence per sample, we decided to take advantage of a PCR amplicon-based platform that we developed to sequence human noroviruses [35]. To this end, we designed a primer that annealed to the first 27 nucleotide of the 5’-end of the sapovirus genome, which is highly conserved among strains, and together with a poly-T primer, would allow full-length viral RNA genome amplification (Figure 1a,b). The amplicons generated would serve as template for NGS analyses. The system showed successful amplification in 15 out of 19 sapovirus-positive samples tested. Representative GI, GII and GV amplicons are shown in Figure 1c. We successfully sequenced 14 full-length sapovirus genome amplicons (≥ 7402 nucleotide) from the archival samples with an average depth of 6026–22516 reads per nucleotide position (Table S2, Figure 1d). The viruses sequenced presented the following genotypes: GI.1 (*n* = 5), GI.2 (*n* = 1), GII.1 (*n* = 7), and GII.4 (*n* = 1) (Figure 2). The amplified GV virus was omitted from the analyses, as it was previously sequenced (GenBank accession MK291480). One limitation of this system is the acquisition and/or selection of mutations during the RT-PCR amplification step. We have developed a similar system for noroviruses [35], and found that RT-PCR amplification of 1 × 10^5^ genome copies of a synthesized norovirus RNA (length: 7550 nucleotide) resulted in ≤10 substitutions/indels arising at ≤12% of all reads, with a depth of coverage ≥1000 (Tohma et al. unpublished results). Despite the ≤12% false-positives derived from RT-PCR, the robustness of this system derived from the high depth of coverage, as compared with the metagenomic approaches that provides low depth of coverage [28]. Additionally, this system provides the opportunity of analyzing multiple samples per NGS run, without compromising the depth of coverage, and thus reducing the cost for large studies. This could be useful for intra-host analyses, as well as to provide accuracy for the consensus genome assembly [28,35].

**Figure 3:**
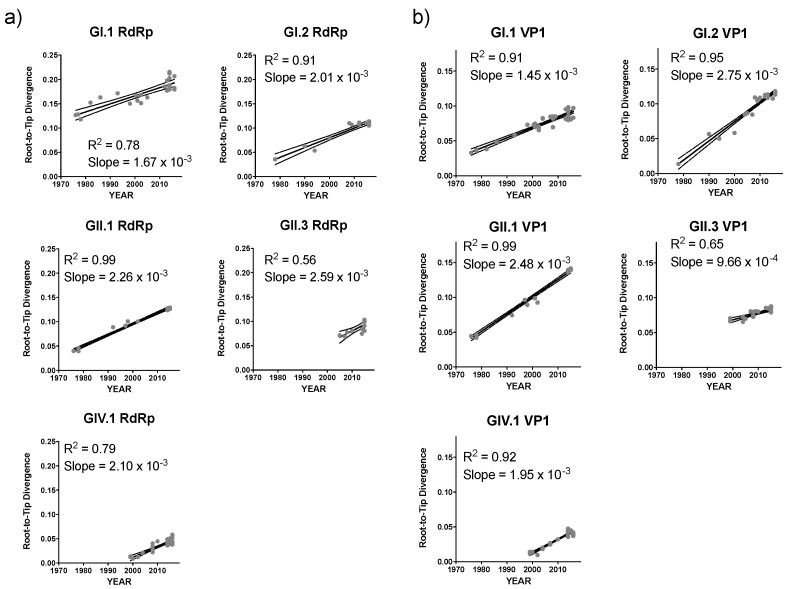
The time-ordered genetic divergence pattern of the RdRp- and VP1-encoding nucleotide sequences of major sapovirus genotypes. Root-to-Tip linear regression analyses were performed to investigate the association between genetic divergence of the (**a**) RdRp- and (**b**) VP1-encoding nucleotide sequences and collection years. The x-axis indicates the collection year and the y-axis shows the root-to-tip divergence on the maximum-likelihood tree. Linear lines indicate the best-fit linear regression of root-to-tip divergence, based on the collection years. Strains are represented by grey dots.

**Figure 4:**
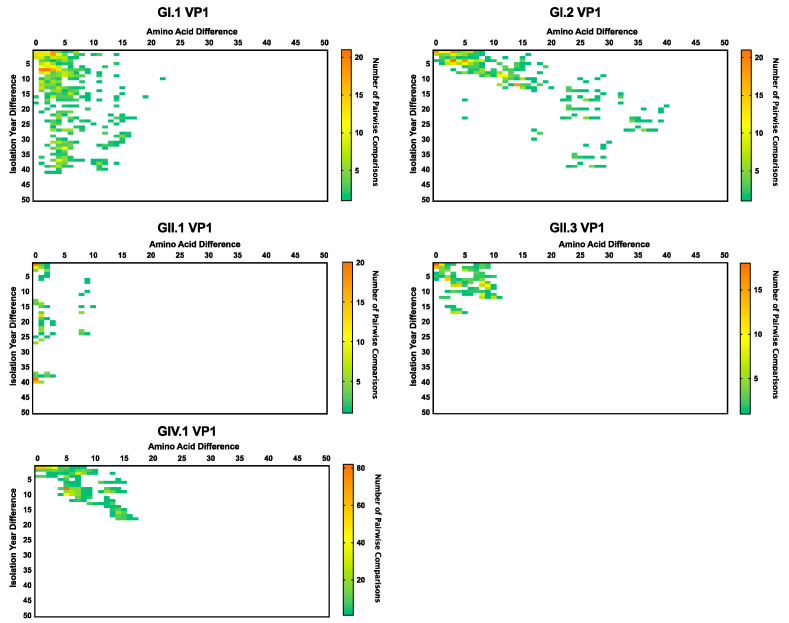
Temporal amino acid diversity patterns of VP1 capsid protein from major sapovirus genotypes. Comparison of patterns reveal differences in VP1 diversification. Over a 40-year period, GI.1 strains differ by 15-20 amino acids, whereas GI.2 strains differ by up to 40 amino acids. The heat map represents the number of pairwise comparisons among available sequences, red being the highest and green the lowest number of pairwise comparisons.

**Figure 5:**
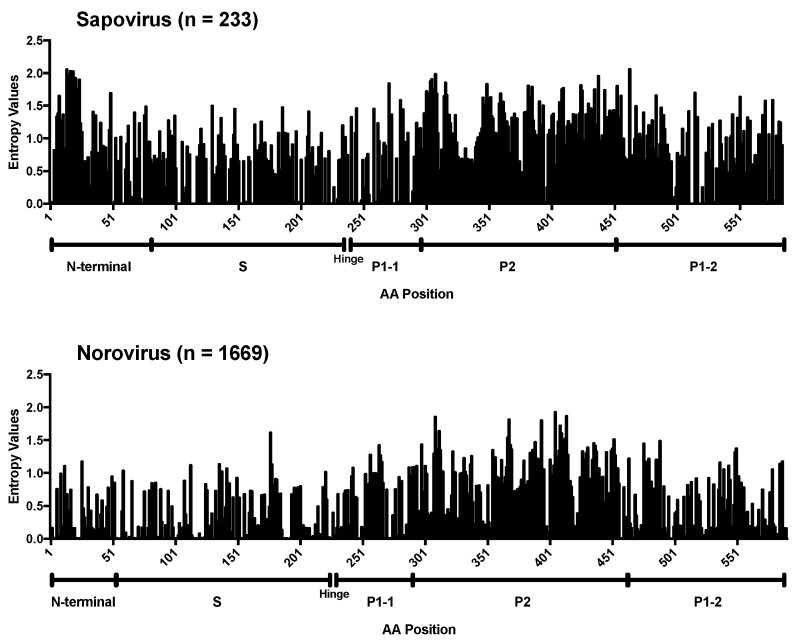
Conservation analyses of the VP1 capsid protein from sapovirus. Shannon entropy values were calculated to quantify the amino acid variation for the capsid protein VP1 from human sapovirus GI, GII, GIV, and GV strains. Sapovirus dataset includes human sapovirus GI, GII, GIV, and GV strains (*n* = 233). Norovirus dataset includes human norovirus GI and GII strains (*n* = 1669). Position of amino acid residues was based on the multiple sequence alignments including indels. Structural domains were assigned based on the sapovirus strain GI.1/Mc114 (AY237422) [3] and norovirus strain GI.1/8FIIa (M87661) [49].

To gain insight into the mechanisms of sapovirus evolution, we mined all full-length (or nearly full-length) capsid (VP1), partial viral RNA-dependent RNA polymerase (RdRp), and full-length (or nearly full-length) genomes of sapoviruses available in the public repositories. We were able to retrieve over 237 sequences from unique human sapoviruses, 125 corresponding to full-length (or nearly full-length) viral genomes, 219 corresponding to VP1, and 152 corresponding to RdRp. The majority of the public sequence data corresponded to strains detected since 2005 (186/237), and only three sequences corresponded to viruses (genotype GI.1) detected in the 1980s (Table 1). Thus, the viruses sequenced in this study provided the oldest sequence information for human sapoviruses. This is very important, as the lack of sequence information on historical viruses could lead to incorrect interpretation of the data [45], e.g., that the “oldest” sapovirus strain, GIII/Cowden/1979, detected in porcine could be the common ancestor of all sapoviruses [46]. The cleavage sites and size of the proteins from full-length genome dataset were predicted based on multiple alignment with a GII.2/Mc10/00 (AY237420) strain [47]. The cleavage sites on NS1/NS2 and NS6-NS7/VP1 were completely conserved, while others presented variation by genotypes (Table 2). The size of the proteins by genotypes are summarized in Table 3. Nonstructural and VP2 proteins showed a similar number of residues, while VP1 proteins showed a difference in its size (range 549–569 amino acids). To investigate the evolutionary pattern of human sapoviruses, we conducted phylogenetic and sequence analyses from five genotypes (GI.1, GI.2, GII.1, GII.3, and GIV.1) that presented ≥20 sequences of VP1 and/or RdRp. All five genotypes showed limited intra-genotype diversification, with mean nucleotide p-distance of 0.034–0.089 (RdRp) and 0.033–0.069 (VP1), and strong clock-like signals (evidenced by root-to-tip linear regression *R*^2^ ≥ 0.56 for RdRp and *R*^2^ ≥ 0.65 for VP1, Figure 3a,b, respectively), with a rate of nucleotide evolution ranging from 1.32 × 10^−3^ to 3.38 × 10^−3^ nucleotide substitutions/site/year (Table 4). The accumulation of amino acid mutations in VP1 was detected for GI.2 and GIV.1 viruses (≤40 amino acid mutations), while minimal changes, ≤5 amino acid mutations over 20 years, were observed in VP1 from GI.1, GII.1, and GII.3 viruses (Figure 4). Despite showing accumulation of mutations, GI.2 and GIV.1 sapoviruses showed only <7% within-genotype differences in VP1 in last 20–40 years (Figure 4). Overall diversity within the VP1 protein showed that N-terminal region and P2 subdomain are the two of the most variable regions (Figure 5) [48]. This is in contrast to human noroviruses (Figure 5) and other caliciviruses [48], which present a conserved N-terminal region and a highly variable P2 subdomain.

**Figure 6:**
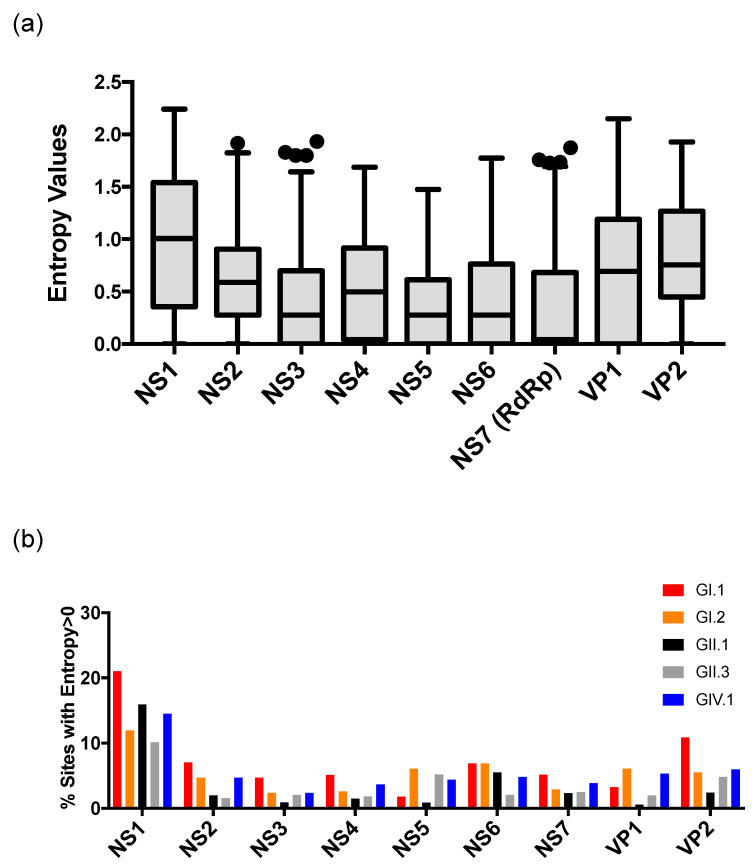
Genome conservation analyses of human sapovirus. (**a**) Shannon entropy values were calculated to quantify the amino acid variation for each site in the nonstructural and capsid proteins in ORF1 and 2 from human sapovirus GI, GII, GIV, and GV strains. Only the sequences with complete length available were included. Boxes represent 25th and 75th percentiles, and whiskers represent 1.5× interquartile ranges from the box. Outliers outside the whiskers were shown by circles. (**b**) Shannon entropy values were calculated for major sapovirus genotypes, and summarized as a ratio (%) of sites with entropy > 0 (i.e., sites with ≥1 mutation(s)) at each of the proteins. Genomic regions were predicted based on the sapovirus strain GI.1/Manchester (X86560) [1].

When analyzing the full-length ORFs of all the human sapovirus strains, amino acid mutations were equally distributed among the proteins, but were relatively higher at the NS1, followed by VP1 and VP2 proteins (Figure 6a, *p* < 0.05 in NS1 vs. all the other proteins except VP2, *p* < 0.05 in VP1 vs. NS1–7, and VP2 vs. NS2-7, from a one-way ANOVA with Tukey’s post-hoc multiple comparison test). The ratio of sites with entropy > 0 (i.e., sites with ≥1 amino acid mutation(s)) was calculated for each protein from the five genotypes analyzed here. NS1 still presented the higher number of mutations at genotype level (Figure 6b). GI.2 and GIV.1 viruses presented a slightly higher number of mutations on VP1 as compared with other genotypes, as was expected from their evolving pattern (Figure 4), but, overall, mutations were very limited. 

The contrast on the evolutionary dynamics between norovirus and sapovirus is noteworthy. While different norovirus genotypes could present multiple variants [35]; sapoviruses showed limited diversification within the genotypes in both nucleotide and amino acid level (Figure 3 and Figure 4), and none of them presented defined variants [1]. In addition, noroviruses presented a single dominant genotype, GII.4, that showed the chronological-emergence of variants; however, no globally dominant genogroups/genotypes has been reported in sapoviruses [50,51,52,53,54,55,56,57]. Thus, although the sapovirus is prevailing in low- and middle-income countries [56,58], limitations on their diversification might restrict their overall prevalence in the human population, as compared to norovirus [52,57,59,60,61].

**Figure 7:**
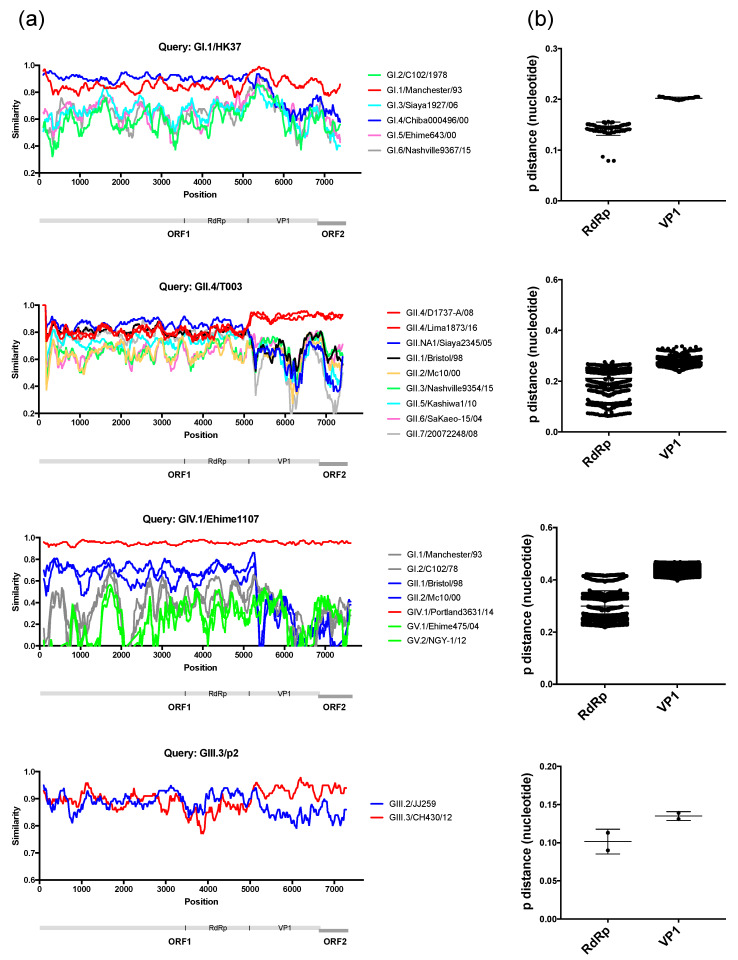
Genomic analyses of putative recombinant sapoviruses present limited evidence of recombination at the RdRp-VP1 boundaries. (**a**) Site-by-site nucleotide similarity analyses of four strains (GI.1/HK7 [MN794208], GII.4/T003 [MN794218], GIV.1/Ehime1107 [DQ058829] [6], and GIII.3/p2 [KX688107] [9]) that presented differences in the phylogenetic clustering when using RdRp- or VP1-encoding regions. (**b**) Inter-genotype/genogroup nucleotide substitution differences among the two genomic regions; RdRp and VP1. The VP1-encoding region presented a higher number of substitutions between the different genotypes/genogroups. Note the lack of both parental strains for the putative recombinant strains.

**Figure 8:**
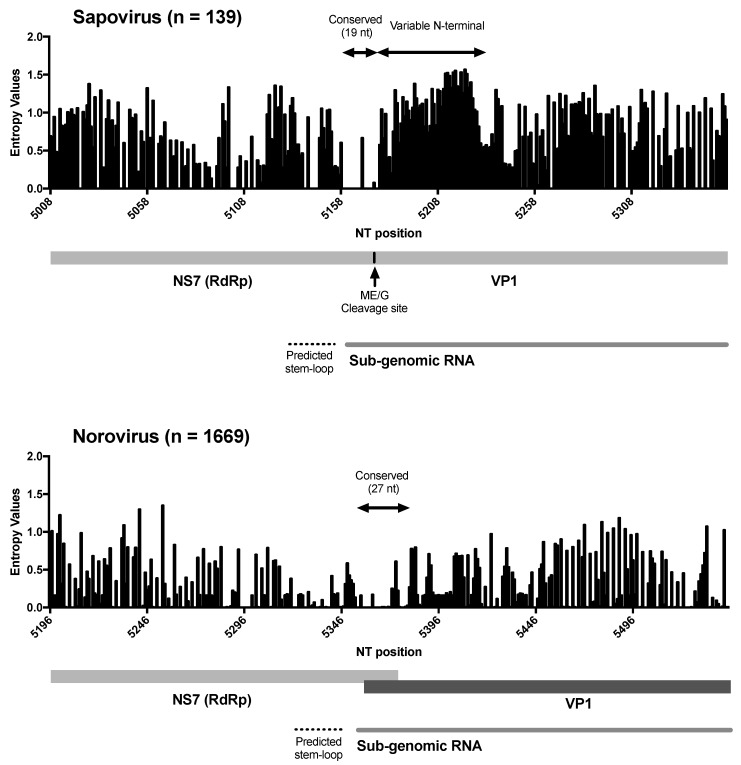
Variation at the RdRp-VP1 boundaries could explain limited recombination in human sapovirus. Shannon entropy values were calculated to quantify the nucleotide conservation for each site in the RdRp-VP1 boundaries. Sapovirus dataset includes human sapovirus GI, GII, GIV, and GV strains (*n* = 139), and norovirus dataset includes human norovirus GI and GII strains (*n* = 1669). Position of nucleotides was based on the multiple sequence alignments, including indels. Stem-loop sequences on predicted sub-genomic RNA promoter and sub-genomic RNA was determined based on analyses from Simmonds et al. [73].

Differences in the phylogenetic clustering was detected between RdRp and VP1 sequences for two of the viruses, HK37/Hong Kong/1977 and T0003/Tunisia/1976, that we sequenced (Figure 2), suggesting potential recombinant strains. We analysed the similarity across the genome of those viruses and representative strains from each of the clusters, and noticed the lack of substantial cross-similarity of the putative recombinant strains with potential parental strains (Figure 7a). We then analysed other recombinant strains reported in the literature [6,9], and found similar patterns on their similarity across the genome with the putative parental strains (Figure 7a). Thus, for example, the GIV strain Ehime1107, which was previously described as recombinant [6], shows the highest similarity (~68% in average) with GII strains at NS proteins, but major differences (0–40%) in the similarity of VP1 and VP2 with all strains (Figure 7a). Moreover, the GIII.3 strain p2, isolated from a piglet and also reported as a recombinant [9], showed high similarity with both parental strains (GIII.2/JJ59 and GIII.3/CH430) at NS proteins, but differences on the similarity at VP1 and VP2 against those parental strains (Figure 7a). Because of the recurrent lack of both parental strains, we examined the inter-genotype/genogroup nucleotide substitution differences among the two genomic regions, i.e., RdRp and VP1, and found that the VP1-encoding region presented a higher number of nucleotide differences between the different genotypes/genogroups (Figure 7b). These differences on the number of substitutions and the lack of substantial cross-over of those putative recombinant strains suggest that recombination events reported for sapoviruses might be the result of differential phylogenetic clustering. False-positives of recombination derived from discordance of tree topologies among genes were similarly addressed in the phylogeny of mammals [62], animal mitochondrial DNA [63], influenza viruses [64], as well as tick-borne encephalitis viruses [65]. Recombination between the RdRp- and VP1-encoding genes has been extensively shown for noroviruses, and proposed to play a major role in the emergence of novel strains [14,66,67]. In sapoviruses, the RdRp and VP1 proteins are encoded in the same ORF1, while in noroviruses, those proteins are encoded by two different ORFs [15]; ORF1 and ORF2, respectively. The high frequency of RdRp-VP1 recombinant norovirus strains has been explained by the fact that the ORF1/2 junction region is highly conserved across norovirus strains, which facilitates template switching between the genomic and/or sub-genomic RNA that encodes VP1 and VP2 [67]. However, while a sub-genomic RNA that includes VP1- and VP2-encoding regions has been detected for sapovirus [68], the RdRp-VP1 junction region of sapovirus showed less conservation compared to noroviruses. These differences were mostly present at the N-terminal encoding region of VP1, as the 5’ end of the sub-genomic RNA was highly conserved in both viruses (Figure 8). Although most DNA and positive-stranded RNA viruses are regarded as prone for recombination [69,70], restrictions on recombination due to differences in the replication mechanisms have been reported for different viruses. Thus, a very low signal for homologous recombination was detected for negative stranded RNA viruses [71] and some positive stranded RNA viruses, e.g., West Nile viruses and tick-borne encephalitis viruses [72]. Therefore, differences in genome organization, the less-conserved RdRp-VP1 boundary, and/or replication mechanisms could limit diversification of sapoviruses by means of RdRp-VP1 recombination. While recombinant and putative parental sapovirus strains have been reported for multiple strains [8,11,12], parental donors for viruses have not yet been detected in nature in many cases [6,7,9]. Further surveillance and phylogenetic analyses will be needed to establish whether recombination is a major driver of sapovirus evolution. 

This study has filled historical gaps in the sapovirus sequence database, as well as provided a genome sequencing platform that can be easily adapted and implemented toward the studies of virus evolution, intra-host dynamics in chronically infected individuals, tracking, traceback and the intervention of clinical and foodborne/waterborne outbreaks of illness due to sapovirus [74]. Sapoviruses present limited intra-genotype diversification by means of amino acid mutations over 20–40 years. Moreover, we presented evidence that human sapoviruses may be less prone to recombination at the RdRp-VP1 boundary as compared to the noroviruses. Understanding the mechanisms of human sapovirus diversification would provide valuable information on the natural history of sapovirus infection that could be used to develop better strategies of prevention and control. 

## References.

## Figures and Tables

**Table 1:** The number of sapovirus sequences newly obtained or retrieved from public repositories for this study, for each genotype on the corresponding year of their detection.

Year	GI.1	GI.2	GI.3	GI.4	GI.5	GI.6	GI.7	GII.1	GII.2	GII.3	GII.4	GII.5	GII.6	GII.7	GII.8	GII.NA1	GIV.1	GV.1	GV.2
1976	1 *							5 *			1 *								
1977	1 *																		
1978	3 *	1 *						2 *											
1982	2																		
1986	1																		
1990		1							1										
1992								1											
1993	1																		
1994		1																	
1995																		1	
1997								1											
1998	1							1											
1999									1	2							3		
2000		1		1	2	1			1			1					2		
2000/01	1																		
2001	2		1					1										1	
2002								1									1		
2002/03	2																	1	
2003											1							1	
2004	2	1		1			1			2			1				2	1	
2005	1	3		1		2				2						1			
2006			1		1				1										
2007	1	1								1							13		
2008	7	1	1			1	1			5	1		2	1	1	1	7		
2009	1	2								1					2				
2010		1								2		1					1	1	
2011											2								
2012	1	5						1			1								1
2013	4	8							1	1									
2014	8							4	2	2		1			3		10	2	
2015	2	1				1		4	1	7		3			1		5	5	
2016	3	10							1		1	1			3		7		2
2018										1									
Total	45	37	3	3	3	5	2	21	9	26	7	7	3	1	10	2	51	13	3

* Sequences obtained in this study.

**Table 2:** Number of sequences by patterns of cleavage sites on the ORF1-encoding proteins.

Genotype	NS1/NS2	NS2/NS3				NS3/NS4			NS4/NS5			NS5/NS6-NS7			NS6-NS7/VP1
	E/G	E/A	E/G	E/S	Q/G	Q/A	Q/G	Q/S	E/A	E/G	Q/A	E/A	E/G	E/S	ME/G
GI.1	23				23	23			23			8	8	7	23
GI.2	11				11			11	10	1		9	2		11
GI.3	2				2			2	2				2		2
GI.4	1				1	1			1			1			1
GI.5	1				1			1	1				1		1
GI.6	3		3				3		3			3			3
GI.7	1				1	1				1		1			1
GII.1	17				17		17		17			17			17
GII.2	6				6		6		6			6			6
GII.3	12				12		12		12			11			12
GII.4	5				5		5		5			5			5
GII.5	6				6		6		6			6			6
GII.6	2				2		2		2			2			2
GII.7	1				1		1		1			1			1
GII.8	8				8		8			8		8			8
GII.NA1	2				2		2		2			2			2
GIV.1	27				27		27		27			27			27
GV.1	8	8						8			8	8			8
GV.2	3			3				3			3	3			3

**Table 3:** Length of nonstructural and structural proteins ^†^.

Genotype	ORF1							ORF2
	NS1	NS2	NS3	NS4	NS5	NS6-NS7	VP1	VP2
GI.1	68	256	341	274	114	668 ^*^	559	166
GI.2	67	256	341	274	115	668	569	164
GI.3	68	256	341	274	114	668	564	166
GI.4	68	256	341	274	114	668	559	166
GI.5	68	256	341	274	114	668	565	166
GI.6	67	256	341	274	114	668	563	166
GI.7	67	256	341	274	115	668	566	166
GII.1	69	256	341	274	115	667	558	167
GII.2	69	256	341	274	115	667	556	167
GII.3	69	256	341	274	115	667	559	167
GII.4	69	256	341	274	115	667	557	167
GII.5	69	256	341	274	115	667	557	167
GII.6	69	256	341	274	115	667	559	167
GII.7	69	256	341	274	115	667	556	167
GII.8	70	256	341	275	115	667	554	167
GII.NA1	69	256	341	274	115	667	557	167
GIV.1	69	256	341	274	115	667	549	168
GV.1	67	257	341	279	123	667	567	167
GV.2	65	257	341	279	123	667	567 ^#^	168

^†^ Length is based on the number of amino acids. ^*^ HM002617/GI.1/Sapporo_MT-2010 strain has 669 amino acids. ^#^ AB775659/GV.2/NGY-1 strain has 569 amino acids.

**Table 4:** Evolutionary rate of the RdRp- and VP1-encoding nucleotide sequences for major five genotypes of human sapoviruses.

Genotype	Mean Substitution Rate, Subs/Site/Year (95% HPD Interval)	
	RdRp	VP1
GI.1	2.25 (1.82–2.69) × 10^−3^	1.38 (1.14–1.65) × 10^−3^
GI.2	3.38 (2.60–4.16) × 10^−3^	1.32 (0.95–1.70) × 10^−3^
GII.1	2.90 (2.33–3.48) × 10^−3^	2.75 (2.21–3.29) × 10^−3^
GII.3	2.82 (1.54–4.11) × 10^−3^	2.31 (1.69–2.94) × 10^−3^
GIV.1	2.82 (2.29–3.36) × 10^−3^	2.00 (1.50–2.51) × 10^−3^

HPD: highest posterior density.

## Supplementary Materials

The following are available online at https://www.mdpi.com/1999-4915/12/5/516/s1, Table S1: Dataset. Table S2: Accession numbers, depth of coverage, and nucleotide length of the consensus sequences from newly obtained sapovirus strains in this study.
